# Emotional Granularity Effects on Event-Related Brain Potentials during Affective Picture Processing

**DOI:** 10.3389/fnhum.2017.00133

**Published:** 2017-03-24

**Authors:** Ja Y. Lee, Kristen A. Lindquist, Chang S. Nam

**Affiliations:** ^1^Industrial and Systems Engineering, University of Wisconsin-Madison, MadisonWI, USA; ^2^Department of Psychology and Neuroscience, University of North Carolina at Chapel Hill, Chapel HillNC, USA; ^3^Industrial and Systems Engineering, North Carolina State University, RaleighNC, USA

**Keywords:** emotional granularity, electroencephalography, event-related potentials, event-related desynchronization and synchronization, affective stimulus processing

## Abstract

There is debate about whether *emotional granularity*, the tendency to label emotions in a nuanced and specific manner, is merely a product of labeling abilities, or a systematic difference in the experience of emotion during emotionally evocative events. According to the Conceptual Act Theory of Emotion (CAT) (Barrett, 2006), emotional granularity is due to the latter and is a product of on-going temporal differences in how individuals categorize and thus make meaning of their affective states. To address this question, the present study investigated the effects of individual differences in emotional granularity on electroencephalography-based brain activity during the experience of emotion in response to affective images. Event-related potentials (ERP) and event-related desynchronization and synchronization (ERD/ERS) analysis techniques were used. We found that ERP responses during the very early (60–90 ms), middle (270–300 ms), and later (540–570 ms) moments of stimulus presentation were associated with individuals’ level of granularity. We also observed that highly granular individuals, compared to lowly granular individuals, exhibited relatively stable desynchronization of alpha power (8–12 Hz) and synchronization of gamma power (30–50 Hz) during the 3 s of stimulus presentation. Overall, our results suggest that emotional granularity is related to differences in neural processing throughout emotional experiences and that high granularity could be associated with access to executive control resources and a more habitual processing of affective stimuli, or a kind of “emotional complexity.” Implications for models of emotion are also discussed.

## Introduction

Imagine a colleague who, when upset at a slight, reports that he feels angry. Now imagine a colleague who when upset at a slight reports that he feels angry, anxious, sad, and disgusted all at once. Whereas the first colleague is having a very specific emotional response, the second is having a much less specific experience—in essence, he is telling you that he just feels unpleasant. The tendency to experience emotions in a highly specific manner is known as ‘emotional granularity’ (Barrett et al., 2001; Demiralp et al., 2012) or ‘emotion differentiation’ (Boden et al., 2013; Kashdan et al., 2015). Growing evidence reveals the importance of this individual difference for emotion regulation (Barrett et al., 2001), decreasing aggressive behavior (Pond et al., 2012), and mental health (Demiralp et al., 2012; Kashdan et al., 2015). Indeed, training individuals to recognize one’s emotions as discrete and specific is a core aspect of many cognitive psychotherapies (Garber et al., 2016). However, no research to date has explored the neural mechanisms underlying individual differences in emotional granularity during affective stimulus processing.

Since individual differences in emotional granularity might manifest as differences throughout the experience of an emotion, we used temporally sensitive electroencephalography (EEG)-based methods to test hypotheses about the psychological and neural mechanisms of emotional granularity. Specifically, we conducted a lab-based EEG study of brain activity in individuals high and low in emotional granularity and measured granularity using an independent measure the night before participants partook in the computerized experiment and EEG recording.

### Emotional Granularity: An Index of Emotional Complexity

Emotional granularity (hereafter, granularity) is the ability to experience emotions in a precise manner and is a kind of ‘emotional complexity associated with emotional and social wellness’ (for reviews, see Lindquist and Barrett, 2008a; Kashdan et al., 2015). For instance, growing research demonstrates that individuals high in granularity, who experience their emotions as discrete and specific, possess greater emotion regulation skills (Barrett et al., 2001) and greater resilience in the face of stress (Tugade et al., 2004) when compared to individuals low in granularity. Greater granularity is also associated with less alcohol abuse in young adults (Kashdan and Ferssizidis, 2010), less aggressive behavior in anger-inducing situations (Pond et al., 2012), and an ability to prevent emotions from biasing moral judgments (Cameron et al., 2013). By contrast, low granularity individuals, who experience their emotions as more diffuse and general, are more likely to hold diagnoses of psychopathologies ranging from borderline personality disorder (Suvak et al., 2011), to major depression (Demiralp et al., 2012) to anorexia nervosa (Selby et al., 2014).

### Hypothesized Mechanisms of Granularity

Despite important work demonstrating the implications of granularity, no research to date has assessed its psychological or neural mechanisms. It is proposed that one psychological mechanism underlying differences in granularity are differences in individuals’ use of concept knowledge about emotion to make meaning of their affective state in a given situation (Lindquist and Barrett, 2008b; Kashdan et al., 2015). Concept knowledge is what a person knows about the categories of ‘anger,’ ‘disgust,’ ‘fear,’ etc. and is acquired in part via prior experience and in part by language (Lindquist, 2013; Lindquist et al., 2015a). According to the Conceptual Act Theory (CAT) of emotion (Barrett, 2006; Lindquist, 2013), an individual experiences an emotion when he or she makes meaning of his or her current affective state using this knowledge. In this view, emotions are ‘conceptual acts’ (Barrett, 2006, 2009) that require the integration of external sensations (visual, auditory, tactile sensations, etc.) and internal sensations (pleasant vs. unpleasant and highly vs. lowly aroused feelings in the body) with concept knowledge. For instance, a person might experience an unpleasant, highly aroused feeling as fear when knowledge about the concept ‘fear’ is relatively more accessible than knowledge about ‘anger’ or other emotions (Lindquist and Barrett, 2008a).

If granularity is related to use of conceptual knowledge, then there may be at least two separate mechanisms that contribute to individual differences in granularity. First, we hypothesize that the ability to experience one’s emotions as discrete and specific, as occurs in high granularity individuals, is contingent on the complexity of someone’s conceptual knowledge about emotions (Lindquist and Barrett, 2008a,b; Kashdan et al., 2015). Individuals who have previously encoded more specific concept knowledge about, say, the features and situations that fear occurs in, will be more likely to categorize and thus experience an unpleasant affective state as fear in a threatening context.

Second, we hypothesize that separate from the content of a person’s conceptual knowledge, his/her ability to wield that conceptual knowledge in the moment will impact granularity (Barrett et al., 2004; Lindquist and Barrett, 2008b; Kashdan et al., 2015). A person’s ability to wield concept knowledge—that is, to access and flexibly use conceptual knowledge—to make meaning of his/her affective experience is ultimately limited by executive control (Barrett et al., 2004; Lindquist and Barrett, 2008a,b; Kashdan et al., 2015). Executive control allows a person to simultaneously access on-going affective feelings and concept knowledge from long-term memory. It is hypothesized that executive control is related to an individual’s working memory capacity (WMC), which governs his/her ability to retrieve information from long-term memory and to inhibit and select between competing sources of semantic information (Engle, 2002; Barrett et al., 2004). In the case of granularity, greater WMC may allow individuals to hold information about their current affective state in mind while they retrieve emotion concept knowledge (i.e., contextual information, semantic knowledge about emotion categories), facilitating the online categorization of their affective state. In fact, evidence (Barrett et al., unpublished) suggests that people higher in granularity tend to be higher in WMC. In a behavioral study, granularity was assessed over the course of a 28-day experience sampling period in which 82 participants rated their experiences of emotion adjectives (*sad, nervous, angry*, and *guilty*) 10 times per day on a palm-top computer. These ratings were then used to compute person-correlations (*p*-correlations; Barrett et al., 2001) that indexed the relatedness of the emotion adjectives within each person, across ratings over time. In a subsequent laboratory session, participants completed several different measures of WMC including standard operation span and reading-span measures popularized by Rosen and Engle (1997) and a novel working memory task for emotional information (E-SPAN). Confirmatory factor analysis indicated that the three measures tapped a single WMC construct and so the measures were thus combined into a single index of WMC that was used as a predictor of participants’ level of granularity. As predicted by the CAT, individuals who were higher in WMC were more granular in daily life: that is, they were less apt to simultaneously say that they were *sad, nervous, angry*, and *guilty* at the same time across multiple sampling instances and instead used emotion adjectives specifically and distinctly at different points throughout the day to describe their experiences. Although preliminary, these findings suggest that individual differences in participants’ ability to wield conceptual knowledge, as mediated by cognitive abilities such as WMC, play an important role in granularity.

Despite this initial behavioral evidence, very little research to date has explicitly explored the online use of these or other possible mechanisms of granularity. One means of examining the mechanisms of granularity is to record the neural activity that is related to individual differences in granularity as individuals experience emotions in real time. No studies to date have specifically measured and modeled the relationship between emotional granularity and neural activity. However, neuroimaging meta-analyses of emotional experiences are instructive as to which neural processes might be predicted, as are the few neuroimaging studies that explicitly examine functional brain activity as it relates to constructs indexing other forms of emotional complexity.

Consistent with the hypothesized mechanisms of granularity, meta-analyses of the neuroimaging literature derived from functional magnetic resonance imaging and positron emission tomography studies (Kober et al., 2008; Vytal and Hamann, 2010; Lindquist et al., 2012; Wager et al., 2015) demonstrate that emotional experiences are associated with increased activity within brain regions related to concept representation and use. That is, brain regions associated with representing concept knowledge, and brain regions associated with the executive control resources necessary for accessing and using conceptual knowledge all generally show consistent increases in activity across emotional experiences (for meta-analyses see Kober et al., 2008; Vytal and Hamann, 2010; Lindquist et al., 2012; Wager et al., 2015). Specifically, there is consistent increase in activity within a distributed network associated with the representation of concepts (i.e., the semantic system; dorsomedial prefrontal cortex, posterior cingulate cortex, lateral temporal cortex, anterior temporal lobe; Binder et al., 2009) across experiences and perceptions of emotion (see Figure 2 in Lindquist et al., 2015b for overlap between regions consistently involved in semantics and emotion). Furthermore, there is increased activity within a set of brain regions involved in executive control and WMC that aid in the retrieval and use of those concepts (i.e., ventrolateral prefrontal cortex, dorsal anterior cingulate cortex; dACC; Thompson-Schill et al., 1997; Miller and Cohen, 2001; Badre et al., 2005; Grindrod et al., 2008) during emotional experiences and perceptions.

Although there are no studies specifically examining whether individuals high in granularity draw on these regions more than individuals low in granularity, a study assessing the neural correlates of a similar construct, “emotional awareness,” suggests that individuals higher in emotional awareness draw on a brain region involved in executive control more than individuals lower in emotional awareness. A positron emission tomography study revealed that individuals who score higher on the Levels of Emotional Awareness Scale (LEAS) (Lane et al., 1990) have greater activity within the dorsal anterior cingulate cortex (dACC) while watching emotional videos and recalling emotional experiences (Lane et al., 1998) than do individuals who score lower on the LEAS. The LEAS assesses people’s propositional knowledge about the emotions that they and others would feel in certain hypothetical situations. Like granularity, the LEAS is considered a measure of emotional complexity (although the two are not correlated, they might both be indicators of the broader construct of emotional complexity; see Lindquist and Barrett, 2008b for a review). Given that the dACC is broadly implicated in executive control and response selection (for reviews see, Holroyd and Yeung, 2012; Shenhav et al., 2016), individuals higher in levels of emotional awareness, like those high in granularity, may more characteristically draw on WMC and executive control to select amongst competing sources of conceptual knowledge during emotional experiences.

A lesion study (?) is also suggestive that regions associated with concept use, WMC, and executive control are related to greater levels of emotional complexity. Barbey et al. (2012) used voxel-based lesion-symptom mapping to identify lesion sites associated with deficits in emotional intelligence across a database of 152 lesion patients. Emotional intelligence was measured via the Mayer Salovey Caruso Emotional Intelligence Test (MSCEIT) (Mayer, 2002), which is a performance-based measure that assesses participants’ ability to perceive emotions in themselves and others, communicate feelings, understand feelings, and manage feelings. Again, although they measure different things, the MSCEIT and granularity are conceptually related and may together be indicative of greater emotional complexity. Critically, consistent with the predictions of the CAT, lower MSCEIT scores were associated with lesions in a distributed set of frontal, temporal, and parietal regions also associated with semantic representation and use, WMC, perceptual organization and processing speed. In particular, lesions within regions such as the ventrolateral prefrontal cortex previously mentioned to be associated with semantic retrieval and executive control were predictive of low MSCEIT scores in this patient sample.

Although these functional neuroimaging and lesion-based studies are suggestive, they did not specifically assess granularity. Nor are these spatially sensitive measures necessarily the best means of testing hypotheses about the mechanisms of granularity. The CAT hypothesizes that representations of affect and the use of conceptual knowledge co-act during the experience of emotion (Barrett, 2006; Lindquist et al., 2012; Lindquist, 2013). Affect does not necessarily precede conceptualization in a linear sequence; rather, the two iteratively shape and constrain one another during a process of constraint satisfaction (Lindquist and Barrett, 2008a; Lindquist et al., 2012). As a result, brain activity during discrete, highly granular emotional experiences will reflect activation of both early neural processes related to affect, as well as both early and later processing related to access to and use of concept knowledge. Fortunately, EEG can address questions about the mechanisms of granularity by capturing activity throughout an emotional experience in a temporally sensitive fashion (e.g., as fast as 1ms after the onset of an emotional stimulus).

No studies to date have focused on the temporal dynamics of neural activity associated with emotional granularity. Thus, our hypotheses about the temporal dynamics of neural activity associated with granularity are based exclusively on theory and the existing EEG literature linking certain ERP components and frequency domains to the psychological processes of interest (see **Table 1**). Under the CAT, high granularity individuals would display greater brain electrophysiological responses consistent with the use of concept knowledge and WMC throughout the timeframe of an emotional experience. We operationalize emotion experience here as the timeframe during which participants are processing an affective stimulus. We make specific predictions for specific event-related potential (ERP) and event-related desynchronization/synchronization (ERD/ERS) outcomes below.

**Table 1:** Functionality of electroencephalography (EEG) methods.

Method	Temporal resolution	Measure	Span of interest	General functionality		Hypothesized emotion-related functionality
ERP	Higher (as low as 1 ms)	Amplitude fluctuations evoked by stimulus	Short (∼1 s)	Early (e.g., P1, N1, N170)	Perceptual feature extraction	Early selective attention to affective stimuli
				Middle (e.g., P2, N2)	Executive control, selective attention	Retrieving concept knowledge for evaluation of affective state
				Late (e.g., P3, N4, Late Positive Potential)	Semantic processing, memory, evaluation	Using concept knowledge for evaluation of affective state

ERD/ERS	Lower [few multiple of ERPs (Knösche and Bastiaansen, 2002)]	Decrease or increase of induced power in given frequency band	Long (1 s∼)	Activation or suppression of cortical activity. Alpha band oscillation is related to the access to concept knowledge. Gamma band is associated with processing of affective stimuli.

### Electroencephalography (EEG) Methods to Investigate Emotional Process

The present study used two quantitative EEG methods to investigate brain activity in response to emotional experiences: ERPs and ERD/ERS. Each method has different value in inspecting neural activity and is differentially linked to the psychological mechanisms hypothesized to support granularity. **Table 1** explains the general functionality and hypothesized emotion-related functionality of each method.

#### Event Related Potentials (ERPs)

Event related potential is the electrophysiological measure of neural activity. The sudden onset of a stimulus evokes prominent electrical peaks on the scalp, and these electrical peaks are measured as ERPs (Blackwood and Muir, 1990). Increased ERP amplitude is considered to reflect the increased engagement of cortical brain areas and the psychological processes they correspondingly support. The fluctuation of electrical power is represented in the time domain, and ERP benefits from the high temporal resolution of the EEG method.

Different ERP components are thought to correspond to different psychological processes relevant to the construction of emotion under the CAT (see **Table 1**). The first, early ERP components (∼200 ms) are known to be very sensitive to the perceptual features of a stimulus. The early reactions in posterior regions are not voluntary but very quick, effortless, automatic evaluation processes initiated by the frontal lobes (Comerchero and Polich, 1999). There are ongoing debates about whether high-level visual perception is represented within this time frame, but the consensus is that coarse visual perception happens before 100 ms, with top-down visual processing of stimuli reflected after 100 ms (see Rossion and Caharel, 2011). These early ERPs might thus reflect selective attention to those stimuli that have already been identified for further processing by the visual system (for a discussion of how projections to orbitofrontal cortex shape visual perception, see Barrett and Bar, 2009). For instance, the P1 and N1 are thought to index early visual processing within extrastriate visual cortex, and respond to the composition, color, and spatial frequency of a stimulus (Olofsson et al., 2008). These ERPs also respond selectively to stimuli greater in valence and arousal as compared to neutral stimuli, and are influenced by selective attention (see Olofsson et al., 2008 for a review).

Subsequent ERP components in the middle range (200–300 ms) represent “cognitive control” (Folstein and Van Petten, 2008) processes, a concept that incorporates use of working memory, initiation of behavioral responses and inhibition of pre-potent responses. Given links between granularity and WMC resources involved in emotion concept retrieval and use (Barrett et al., 2004; Lindquist and Barrett, 2008b; Kashdan et al., 2015) we predict that amplitudes of these components will differ based on participants’ level of granularity. For instance, the P2 and N2 in fronto-central and temporo-occipital sites are thought to reflect executive control processes (Codispoti et al., 2006; Bradley et al., 2007). The closely related early posterior negativity (EPN; ∼200–300 ms) over central and temporo-occipital sites is thought to reflect “natural selective attention” to salient stimuli (Dolcos and Cabeza, 2002; Schupp et al., 2004; Olofsson et al., 2008). Critically, these middle ERPs also respond selectively to the arousal content of a stimulus (see Olofsson et al., 2008), consistent with the interpretation that stimuli that warrant attention, especially emotional stimuli, selectively activate these components.

Finally, ERPs in the late range (300 ms∼) are associated with an “informational processing cascade” when attentional and memory processes are engaged to process the meaning of a stimulus (Polich, 2007), and presumably the meaning of a person’s reaction to that stimulus. For instance, P3 has been suggested to be the result of various executive functions such as: content evaluation (Soltani and Knight, 2000), short-term memory storage (Conroy and Polich, 2007), and decision-making (Nieuwenhuis et al., 2005). Consistent with the idea that the P3 relates to information processing, both the valence and arousal content of a stimulus (relative to neutral content) is associated with greater P3 responses (Olofsson et al., 2008). The related midline late positive potential (LPP; >400 ms) is similarly thought to be involved in “motivated attention” and executive control (Hajcak et al., 2009). For instance, in emotion, the LPP has been indicated in emotion regulation when alternate meaning is made of stimuli (i.e., the emotional meaning of stimuli is reappraised) (Hajcak and Nieuwenhuis, 2006; Moser et al., 2006; Foti and Hajcak, 2008). Finally, the N4 is a negative component that is linked to meaning processing, or the integration of meaning extracted from multiple modalities (Kutas and Federmeier, 2011). Insofar as individuals high in granularity are recruiting attentional and executive control resources for categorization of the meaning of affective states to a greater extent than individuals low in granularity, we predict that high granularity individuals will have greater amplitudes within this later range.

We examine ERPs in high and low granularity individuals across several different emotion categories (awe, excitement, fear, and disgust), but we do not have *a priori* hypotheses about how viewing different images normed to induce specific ‘discrete emotions’ will influence ERPs or how granularity will interact with emotion category to influence ERPs. To our knowledge, there is little research examining ERPs for specific discrete emotions, although there is a fair amount of research examining ERPs to the valence (positive vs. negative) and arousal (high vs. low activation) qualities of affective stimuli (see Olofsson et al., 2008 for a review). The functional magnetic resonance imaging literature demonstrates that processes representing valence and arousal, as well as access to and use of conceptual knowledge are generally involved across different discrete emotions (Lindquist et al., 2012; Touroutoglou et al., 2015; Wager et al., 2015). We thus do not predict emotion-specific ERP outcomes nor specific interactions between granularity and emotion category. Thus, any main effects of emotion category or granularity × emotion category interactions observed should be considered exploratory and subject to limited interpretation in the absence of replication.

#### Event-Related Desynchronization and Synchronization (ERD/ERS)

Event-related desynchronization and ERS indicate how much activity within a specific frequency band has been decreased (ERD) or increased (ERS) when an event takes a place. Specifically, ERD indicates that neural generators of a specific frequency are actively desynchronized from others. The result is decreased rhythmic activity, which indicates increased cortical activity. ERS, on the other hand, indicates the opposite activity. Neural generators of a specific frequency are actively synchronized with others, resulting in increased rhythmic activity and decreased cortical activity (Nam et al., 2011). These processes are relevant to the construction of emotion under the CAT of emotion (see **Table 1**) and we predict that ERD/ERS will differ based on participants’ level of granularity.

Event-related desynchronization or ERS is typically measured in different frequency bands, divided into delta (0.5–4 Hz), theta (4–8 Hz), alpha (8–13 Hz), beta (13–30 Hz), and gamma (over 30 Hz) bands. Following Jaušovec et al. (2001) and Jaušovec and Jaušovec (2005), who examined correlates of emotional intelligence as measured by the MSCEIT, we focus specifically on the ERD of the alpha band power. Alpha power has long been associated with most executive control processes, such as working memory and is also associated with selective attention, and perception, more generally. For instance, ERD of alpha oscillation is thought to represent ‘enhanced information transfer’ during creativity tasks as individuals draw on top-down control of information from working memory (Benedek et al., 2011). More relevant to emotion, Jaušovec et al. (2001) and Jaušovec and Jaušovec (2005) assessed the association between alpha ERD and emotional intelligence using the identification of emotions portion of the MSCEIT (Mayer, 2002). They observed less alpha ERD in individuals who were high vs. average in emotional intelligence when individuals were labeling emotions depicted in posed facial expressions. Their conclusion was that, as a form of intelligence drawing on resources typically involved in verbal intelligence, individuals high in emotional intelligence showed greater “neural efficiency” during the labeling of faces. These findings mirror those observed when highly vs. lowly intelligent individuals perform difficult cognitive tasks (Grabner et al., 2006). This logic is consistent with our hypothesis that granularity involves greater access to and use of conceptual knowledge by employing executive control processes such as WMC.

We also examined differences in gamma ERS between highly and lowly granular individuals during affective picture viewing in the present study. Gamma oscillations are related to a broad range of processes such as feature integration, attention, stimulus selection, integration of sensory inputs and sensorimotor activities, movement preparation, and memory formation (Knyazev, 2007) and synchronization is thought to reflect greater cortical processing reflecting these functions.

Because ERD/ERS is computed over a longer time frame than ERPs (> ∼1 s) and thus reflects sustained processing, we predicted that individuals *low* in granularity would have more sustained activity of executive control processes over the course of the entire emotional experience, as reflected by more alpha ERD and more gamma ERS. This result is consistent with the “neural efficiency” account (Jaušovec and Jaušovec, 2005; Grabner et al., 2006) which claims that more intelligent people have less cortical activation during difficult tasks than do less intelligent people. In essence, we predicted that high granularity individuals would require cognitive operations early in an emotional experience to make meaning of affective states as specific emotions, but then require less sustained processing of executive attention resources than lowly granular individuals for the remainder of the emotional experience. Low granularity individuals, on the other hand, would require more sustained processing to make meaning of their affective responses to the affective stimuli. Like Jaušovec and Jaušovec’s (2005) findings, this finding would reflect the idea that highly granular individuals are more ‘emotionally intelligent’ and would be consistent with the idea that granularity is a stable form of emotional complexity (Lindquist and Barrett, 2008a; Kashdan et al., 2015).

As in the case of the ERP analysis, we also examined main effects of emotion category on ERD/ERS and interactions between granularity and emotion category on ERD/ERS, but we do not have *a priori* hypotheses about these outcomes. Thus, any main effects of emotion category or granularity × emotion category interactions observed should be considered exploratory and subject to limited interpretation in the absence of replication.

## Materials and Methods

### Participants

A total of 33 (11 female) participants from a local university participated in the EEG experiment for class credit. The average age was 21.52 years (*SD* = 2.11 years). Thirty out of the 33 participants were right-handed. There were no participants with previous neurological disease. Participants gave their written consent after a detailed explanation of the experiment procedure, which was approved by the University’s Institutional Review Board.

### Apparatus

#### Modified Day Reconstruction Method

All participants completed an online survey designed to assess their degree of granularity the night before completing the lab-based measure of emotional experience and EEG recording. The survey consisted of demographic questions and the modified day reconstruction method (DRM by Kahneman et al., 2004). The survey was anticipated to take approximately 45 min to complete.

When answering the modified DRM, the participants were asked to recall up to five episodes from the morning, five episodes from the afternoon, and five episodes from the evening of the day before the survey (2 days before the experiment). For each episode, they were asked: what they were doing, where they were, and whom they were interacting with. Finally, they indicated to what level (from 0 to 6) they experienced each of 20 emotion categories while they were experiencing the episode. Ten emotion categories were positive (amusement, awe, contentment, excitement, gratitude, happiness, love, pleased, pride, and serenity), and the other 10 were negative (anger, boredom, disgust, dissatisfied, downhearted, embarrassment, fear, gratitude, sadness, and tired) to fully sample the range of affective space. As a result, each participant rated 20 emotions during up to 15 episodes.

Participants’ experience sampling reports were then used to calculate their degree of granularity in daily life. As per the literature, granularity was computed as the co-variation between participants’ use of emotion terms across emotional experiences in the day they were reporting on (Barrett et al., 2001; Demiralp et al., 2012). More specifically, average intraclass correlations (ICCs) were calculated from participants’ self-reports of emotion categories across episodes. ICCs are standardly used to calculate granularity (Tugade et al., 2004; Kimhy et al., 2014). ICCs of positive emotion categories and ICCs of negative emotion categories were separately calculated and averaged for each participant to make a single granularity value. A low ICC value indicates that the participant can differentiate discrete emotional categories and express their emotional experiences with different emotion terms. On the other hand, high ICC value means that the participant use terms interchangeably to communication their emotional state. Thus, the ICCs were subtracted from 1 for ease of interpretation to make higher values correspond to more differentiation, or granularity. The participants’ average granularity was 0.78 (*SD* = 0.16).

#### Affective Picture Stimuli

For the EEG data collection, a total of 50 images (10 images for each of five emotional categories including awe, excitement, fear, disgust, and also neutral control images) were selected from the International Affective Picture System (IAPS by Lang et al., 1999) based on norms from Mikels et al. (2005). Awe, excitement, fear, and disgust were chosen as discrete emotion categories, because these categories are thought to be relatively comparable in terms of arousal in a standard emotional circumplex. Participants saw blocked sets of images that induced awe, excitement, fear, disgust, and neutral, although the specific order of images was randomized within each emotion block.

**FIGURE 1:**
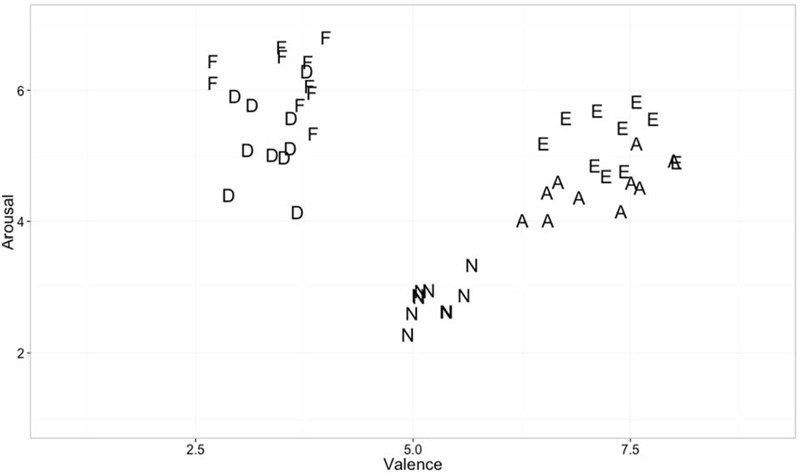
**Valence and arousal of emotional stimuli used in the experiment**.

**Figure 1** shows valence and arousal values of emotional stimuli used in the present experiment. Despite attempting to match stimuli on both valence and arousal, it was impossible to fully do so. The images similar in valence were distinguishable when plotted on awe-excitement space or on fear-disgust space. For example, Awe and excitement image norms did not differ in terms of valence [*t*(18) = 0.80, *p* = 0.43] but differed in level of arousal [*t*(18) = 4.30, *p* < 0.001]. Fear and disgust images were also not different in valence [*t*(18) = 0.98, *p* = 0.34], but differed in level of arousal [*t*(18) = 3.85, *p* < 0.01]. Arousal was also higher for negative stimuli when compared to positive stimuli [*t*(38) = -4.11, *p* < 0.001]. Differences in valence and arousal of stimuli thus cannot be ruled out as contributors to any main effects of emotion category or granularity × emotion category interactions observed.

### Procedure

**FIGURE 2:**
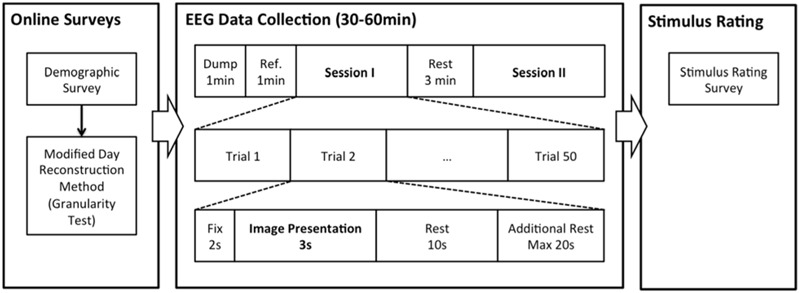
**Procedure of the present experiment**.

**Figure 2** summarizes the overall procedure of the experiment. All participants completed online surveys (modified day reconstruction) on the day before the EEG data collection. EEG data collection was separated into two identical sessions with 50 trials each and a 3 min rest session in between. Signals from the first 1-min were dumped, and the next 1 min of data were used as referencing data and baseline correction. The participant took rest with open eyes during the referencing period. In each trial, a participant viewed an affective image for 3 s after seeing 2 s of fixation cross, and took a rest for 10 s after viewing the image. An additional rest time of 20 s maximum was provided when the participant’s EEG signals did not return to rest (i.e., more than 5% of EEG signals were outside the 2σ of the signal collected during the reference period). After the signal collection was completed, the participant rated each affective image based on their emotional experience during the data collection. In particular, participants sat on the same chair used for the EEG recording and were given survey papers and a pen. The survey papers contained all 50 images presented during the EEG recording. For each image, participants chose one emotional word from neutral, awe, excitement, fear, and disgust, and specified the strength of the selected emotion (on a 0–6 scale).

### Manipulation Check

**FIGURE 3:**
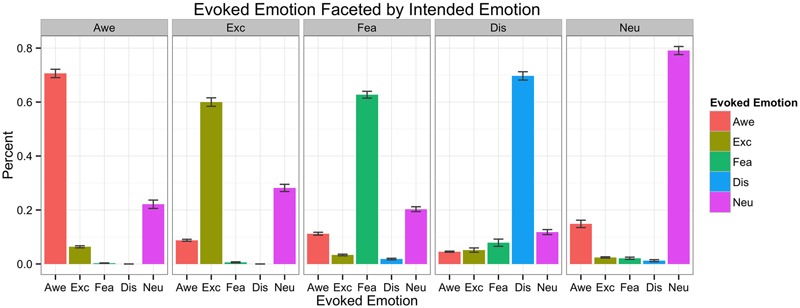
**Intended emotion of stimuli and actually evoked emotions for each intended emotion**.

**Figure 3** shows what percentages of images were successful in eliciting the emotion from Mikels’ et al. (2005) norms in our sample of 33 participants. We also broke down rates of endorsements between highly and lowly granular individuals to ensure that there were not systematic differences between the two groups in their agreement about whether a given stimulus elicited feelings of specific emotions. We observed few differences in highly vs. lowly granular people in terms of mean endorsement of the individual stimuli (whose granularity value was greater than 1 standard deviation above or below the sample mean, respectively). It may seem counter-intuitive that highly and lowly granular people showed similar patterns of endorsement for the awe-inspiring, exciting, fearful and disgusting images. However, granularity is an index of the co-occurrence of multiple same-valence emotions across instances when participants are able to endorse multiple same-valence emotion categories at the same time (e.g., low granularity individuals say they are feeling both awe and excitement at the same time across multiple instances of pleasant emotions). Thus, we would not expect to see systematic differences in a task such as this manipulation check in which participants were asked to choose only a single label using a forced-choice method from a small set (awe, excitement, fear, disgust, and neutral) on one instance.

### Data Acquisition and Signal Processing

The participants were seated in a comfortable chair 50″ in front of the TV monitor that images were to be displayed on. Participants wore an EEG cap embedded with 16 electrodes covering Fp1/Fp2, F7/F8, FC3/FC4, T7/T8, P7/P8, FT7/FT8, P3/P4, C3/C4 areas, based on the modified 10–20 systems of the International Federation (Niedermeyer and da Silva, 2005). Fpz was used as a ground, and left ear lobe was used as a reference. Because the reference was closer to the left hemisphere, the amplitude of ERP on this side might have been decreased. However, as we analyzed four brain regions (i.e., frontal, central, temporal, and posterior) that are symmetric, the effect of this asymmetric reference was minimized. The areas were chosen based on the Brodmann Area (BA), based on a region’s involvement across meta-analyses of the neuroimaging literature on emotion (Kober et al., 2008; Lindquist et al., 2012).

Signal was amplified with a g.USBamp amplifier from g.tec Medical Engineering, band-pass filtered between 0.1 and 50 Hz and digitized at a rate of 256 Hz, using g.tec LabVIEW modules. In this experiment, trials with amplitudes exceeding ±35 μV measured after stimulus onset were excluded from analysis. The threshold 35 μV is an average of 99.7% confident intervals of all participants. As a result, 3.25% of total trials were removed. Each confidence interval was calculated by sampling all data points collected during -200 ∼ 1000 ms epoch and estimating μ ± 3σ. This value was enough to remove the effect of eye blinks that causes voltage over 60 μV. In addition, collected EEG data underwent Common Average Reference (CAR) calculation, where average amplitude of signals from every electrode site was used as a reference.

### Data Analysis

#### Event Related Potential (ERP)

First, we categorized 16 channels into the following four brain regions and averaged the activation of cortex within each region: frontal (Fp1/Fp2/F7/F8), temporal (FT7/FT8/T7/T8), central (FC3/FC4/C3/C4), and posterior (P3/P4/P7/P8). Then, for each participant, 20 epochs for each of four discrete emotions and neutral states were obtained. The length of each epoch was 600 ms, starting from stimulus onset (0 ms). An average of 20 epochs became the representative ERP waveform for the emotion. Then, the 600 ms epoch was separated into 20 time bins (30 ms long each), and the average ERP within each bin was calculated, as binning in narrow bins enhances resolution lost from averaging across participants (Poli et al., 2010).

#### Event Related Desynchronization/Synchronization (ERD/ERS)

Four groups of brain regions (frontal, temporal, central, and posterior) were used for analysis, as in the ERP analysis. To compute ERD/ERS, all trials were band-pass filtered by the frequency band of interest: alpha (8–12 Hz) or gamma (30–50 Hz). Filtered signals were squared and then averaged over the total number of trials to reduce noise. The length of epoch was 4000 ms (from -1000 to 3000 ms). The first 1000 ms interval was the reference, while the next 3000 ms was the interval of interest. The power of 3000 ms was averaged for 10 time bins with length of 300 ms each (Avanzini et al., 2012). Using the standard ERD/ERS calculation (Pfurtscheller and Aranibar, 1979), the quantification of ERD/ERS was carried out in four steps: (1) bandpass filtering for all event-related trials, (2) squaring the amplitude samples to obtain power samples, (3) averaging power samples across all trials, and (4) averaging time samples to smooth data and reduce variability (Pfurtscheller and Lopes da Silva, 1999).

$$\mathrm{ERD},\;ERS=\frac{A-R}{R}\times 100\%$$

*R* is the average power during the reference period (i.e., from -1000 to 0 ms), and *A* is the average power in the interval of interest (i.e., 0–3000 ms). Decrease in the value (i.e., ERD) indicates that there is decrease in power, and increase in the value (i.e., ERS) indicates that there is increase in power. We focused on alpha ERD and gamma ERS.

### Statistical Analysis

We hypothesized that emotional stimulus processing would be influenced by granularity, brain region, and the emotion category evoked by a given image. We used regression models in order to model the continuous nature of granularity scores. First, the averaged ERP or ERD/ERS value was separately analyzed for each time bin. There are three variables and their interactions: granularity, brain region, and emotion category. Granularity is a continuous variable centered around the mean, brain region is a factor variable with four levels (frontal, temporal, central, and posterior) with temporal as a reference, and discrete emotion is a factor variable with five levels (awe, excite, fear, disgust, and neutral) with neutral as a reference.

## Results

### Effects of Granularity and Emotion on ERP Patterns

**FIGURE 4:**
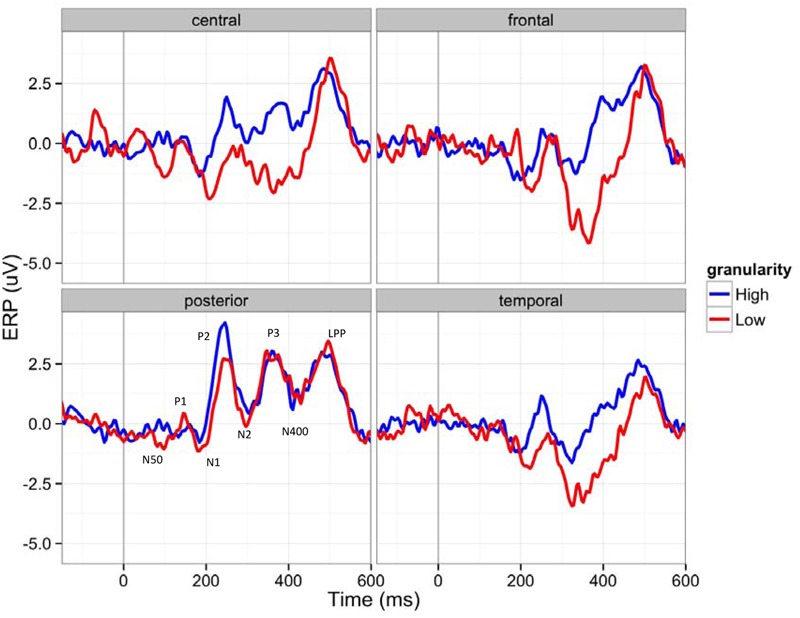
**Event-related potentials (ERP) waveforms of participants with higher (over 1 SD) and lower (under 1 SD) granularity (for demonstration purposes) in four brain regions (C: central, F: frontal, P: posterior, T: temporal): The 150 ms pre-stimulus period was used as the baseline**.

**Figure 4** shows ERP waveforms of highly granular (granularity higher than ‘mean + 1 standard deviation’) and lowly granular (granularity lower than ‘mean - 1 standard deviation’) people in four brain regions: central, frontal, parietal, and temporal. In general, participants showed an early positive peak near 150 ms (P1), a negative peak near 180 ms (N1), a middle positive peak near 240 ms (P2), a negative peak near 300 ms (N2), a positive peak near 350 ms (P3), a negative peak near 400 ms (N400), and a late positive peak near 510 ms (LPP). The amplitude greatly decreased to a negative amplitude after the late positive peak, resembling ERP waveforms reported in other studies (e.g., Costanzo and McArdle, 2013). The main effect of granularity was significant between 60–90 ms (β = 2.01, *F* = 10.03, *p* = 0.0016), 270–300 ms (β = -3.86, *F* = 20.43, *p* < 0.0001), and 540–570 ms (β = 3.29, *F* = 10.72; *p* = 0.0011), when we used alpha level 0.0025 (Bonferroni correction for 20 time bins). As described in **Figure 4**, this led to more negative early ERPs in the lowly granular group, a more negative N2 peak of the highly granular group, and a fast ERP drop after the LPP of the highly granular group.

**FIGURE 5:**
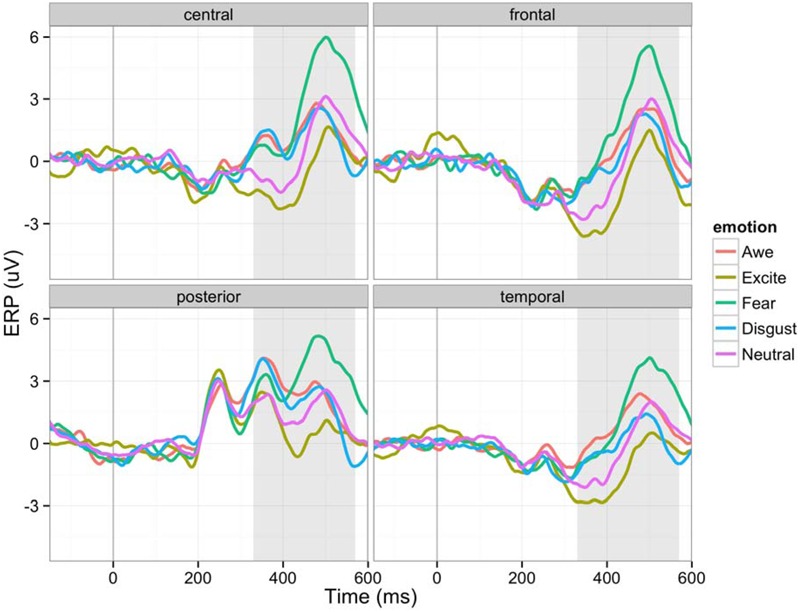
**Event-related potential waveforms for five different emotions in four brain regions (C: central, F: frontal, P: posterior, T: temporal): The 150 ms pre-stimulus period was used as the baseline**.

As shown in **Figure 5**, the main effect of emotion was significant after 330 ms until 570 ms (*p* < 0.0001 in all time bins; *F* = 9.72, *F* = 11.17; *F* = 12.13; *F* = 14.88; *F* = 15.84; *F* = 14.23; *F* = 15.75; *F* = 13.97), indicating that differences between emotion categories emerged only after these middle-to-late ERPs. See **Table 2**. There was no interaction effect between granularity and brain regions or the interaction between emotion and brain regions in any time bin, although there were main effects of brain regions (between 180–390 ms and 480–600 ms).

**Table 2:** Main effects of emotion category on event-related potential (ERP) amplitude.

Time bin	Emo main effect	Awe	Excite	Fear	Disgust
330–360 ms	*F* = 9.72, *p* < 0.0001				β = -1.46, *t* = -4.78, *p* < 0.0001
360–390 ms	*F* = 11.18, *p* < 0.0001	β = 1.07, *t* = 3.57, *p* = 0.0004	β = 0.94, *t* = 3.12, *p* = 0.0019		β = -1.53, *t* = -5.11, *p* < 0.0001
390–420 ms	*F* = 12.13, *p* < 0.0001	β = 1.00, *t* = 3.34, *p* = 0.0009	β = 1.20, *t* = 4.01, *p* < 0.0001		β = -1.69, *t* = -5.65, *p* < 0.0001
420–450 ms	*F* = 14.88, *p* < 0.0001		β = 1.80, *t* = 5.75, *p* < 0.0001		β = -1.89, *t* = -6.04, *p* < 0.0001
450–480 ms	*F* = 15.84, *p* < 0.0001		β = 2.17, *t* = 6.83, *p* < 0.0001		β = -1.73, *t* = -5.44, *p* < 0.0001
480–510 ms	*F* = 14.23, *p* < 0.0001		β = 2.26, *t* = 6.91, *p* < 0.0001		β = -1.14, *t* = -3.49, *p* = 0.0005
510–540 ms	*F* = 15.75, *p* < 0.0001		β = 1.94, *t* = 6.48, *p* < 0.0001	β = -1.43, *t* = -4.79, *p* < 0.0001	
540–570 ms	*F* = 13.97, *p* < 0.0001		β = 1.76, *t* = 5.68, *p* < 0.0001	β = -1.75, *t* = -5.66, *p* < 0.0001	

Although not predicted *a priori*, we found interaction effects between granularity and emotion (summarized in **Table 3**). The results suggest that an individual’s level of granularity had an early- to mid-time frame moderating effect on brain signals responding to the experience of different emotion categories. Given that we did not have *a priori* hypotheses about how granularity would interact with specific emotion categories, we reserve making strong interpretations of these findings, but they suggest that the degree to which a person characteristically draws on conceptual knowledge of emotion might interact with the specific emotion content being experienced. A caveat here is that these effects could be a result of the methods used and should be limited to inferences about emotional picture viewing and may not extend to more ecologically valid contexts (e.g., social emotional interactions in daily life).

**Table 3:** Interaction between granularity and emotion category on ERP amplitude.

Time bin	Gran ^∗^ Emo interaction	Awe	Excite	Fear	Disgust
0–30 ms	*F* = 5.32, *p* < 0.001				β = -4.03, *t* = -3.90, *p* < 0.0001
90–120 ms	*F* = 6.60, *p* < 0.0001		β *=* 4.45, *t* = 3.26, *p* = 0.0012		β = -5.57, *t* = -4.90, *p* < 0.0001
180–210 ms	*F* = 5.34, *p* < 0.001		β = 6.21, *t* = 4.09, *p* < 0.0001		
210–240 ms	*F* = 8.67, *p* < 0.0001		β = 8.74, *t* = 5.15, *p* < 0.0001	β = -6.38, *t* = -3.76, *p* < 0.001	
240–270 ms	*F* = 6.13, *p* < 0.0001		β = 7.03, *t* = 4.32, *p* < 0.0001	β = -5.38, *t* = -3.31, *p* < 0.001	

### Granularity and Emotion Effects on ERD/ERS

**FIGURE 6:**
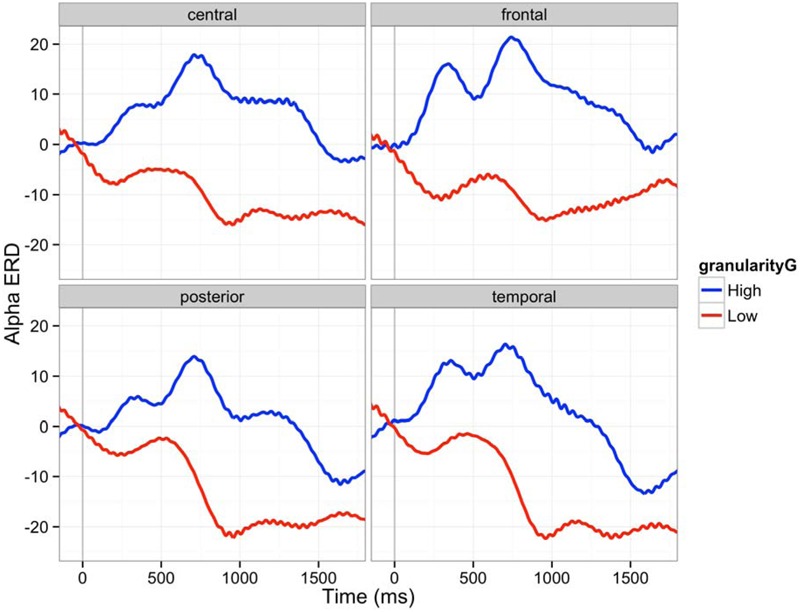
**Alpha ERD (8–12 Hz) in four brain regions (C: central, F: frontal, P: posterior, T: temporal): The 150 ms pre-stimulus period was used as the baseline.** Red line is an average ERD of five participants whose granularity was above one sigma of average granularity. Blue line is an average ERD of four participants whose granularity was below one sigma of average granularity. The 150 ms pre-stimulus period was used as the baseline.

The epoch of this analysis was between stimulus onset and 3000 ms. The epoch was segmented into ten 300 ms time bins. We first analyzed alpha ERD. The power of alpha band (8–12 Hz) decreased (ERD) during the first 600 ms after the stimulus onset as described in **Figure 6**. Lowly granular people particularly showed more ERD, an index of greater cortical activation. Such difference between lowly and highly granular individuals was significant after 900 ms post-stimulus onset and remained significant through 2700 ms (900–1200 ms: β = 45.5, *t* = 5.16, *p* < 0.0001; 1200–1500 ms: β = 48.8, *t* = 6.07, *p* < 0.0001; 1500–1800 ms: β = 40.0, *t* = 5.02, *p* < 0.0001; 1800–2100 ms: β = 26.2, *t* = 3.23, *p* < 0.005; 2100–2400 ms: β = 30.9, *t* = 3.61, *p* < 0.001; 2400–2700 ms: β = 31.6, *t* = 3.83, *p* < 0.0001). We used alpha level of 0.005 to make Bonferroni corrections on 10 time bins. No emotion category main effect or interaction of granularity and emotion category was observed in this frequency band.

**FIGURE 7:**
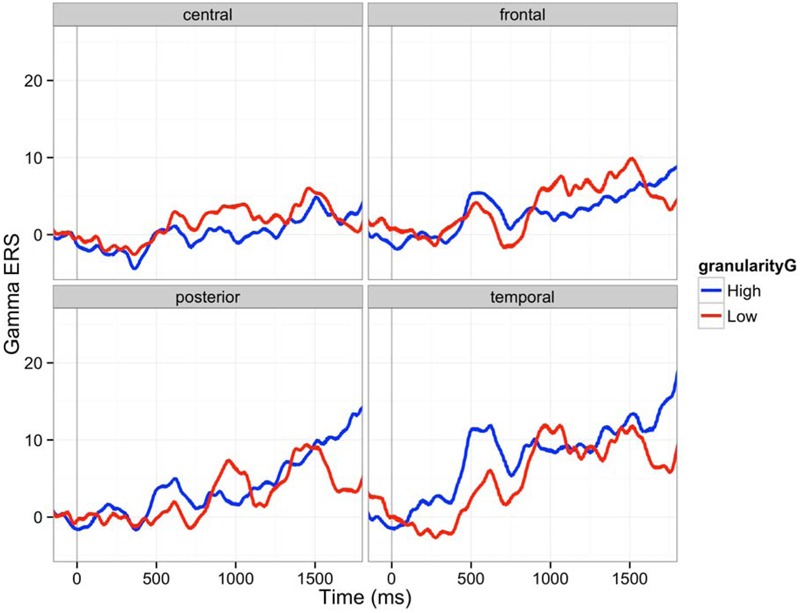
**Gamma ERS (30–50 Hz) in four brain regions (C: central, F: frontal, P: posterior, T: temporal): The 150 ms pre-stimulus period was used as the baseline.** Red line is an average ERS of five participants whose granularity was above one sigma of average granularity. Blue line is an average ERS of four participants whose granularity was below one sigma of average granularity.

Next, we analyzed gamma ERS. Gamma oscillation increased after the stimulus onset (ERS) as shown in **Figure 7**. Contrary to the alpha band, the effect of granularity was minimal, but we found a main effect of emotion category and interactions between granularity and emotion category in gamma band ERS. The main effect of granularity in the gamma band was significant only between 900 and 1200 ms (β = -14.74, *F* = 10.08, *p* < 0.005). Individuals high in granularity tended to show lower ERS compared to the low granular people in these time bins. The main effect of emotion category was significant in all bins except for 0–300 ms, as **Table 4** shows, meaning the power of gamma oscillation was influenced by the emotion category seen over the time of image presentation. The interaction of granularity and emotion category was significant between 300–600 ms and 900–1200 ms, where high granular people showed less ERS while viewing disgust stimuli (β = -38.8, *t* = -4.70, *p* < 0.0001), 900–1200 (β = -35.2, *t* = -3.79, *p* = 0.0002), and also between 1800 and 2400 ms, where high granular people showed less ERS while viewing excitement stimuli (β = -74.4, *t* = -5.46, *p* < 0.0001; β = -64.2, *t* = -4.58, *p* < 0.0001). There was no interaction effect associated with brain regions (main effects exist between 600 and 2700 ms).

**Table 4:** Emotion effect on gamma ERS.

Time bin	Emotion	Awe	Excite	Fear	Disgust
300–600 ms	*F* = 4.86, *p* < 0.001	β = -4.79, *t* = -3.77, *p* = 0.0002	β = 3.92, *t* = 3.09, *p* = 0.0021		
600–900 ms	*F* = 6.04, *p* < 0.0001	β = -3.45, *t* = -2.81, *p* = 0.0051	β = 4.26, *t* = 3.47, *p* = 0.0006		
900–1200 ms	*F* = 10.06, *p* < 0.0001	β = -4.68, *t* = -3.28, *p* = 0.0011	β = 4.75, *t* = 3.33, *p* = 0.0009		β = 4.75, *t* = 3.33, *p* = 0.0009
1200–1500 ms	*F* = 8.64, *p* < 0.0001	β = -5.02, *t* = -3.30, *p* = 0.0010	β = 7.29, *t* = 4.80, *p* < 0.0001		
1500–1800 ms	*F* = 6.09, *p* < 0.0001		β = 7.62, *t* = 4.28, *p* < 0.0001		
1800–2100 ms	*F* = 12.93, *p* < 0.0001		β = 14.66, *t* = 6.99, *p* < 0.0001		
2100–2400 ms	*F* = 16.68, *p* < 0.0001		β = 16.68, *t* = 7.70, *p* < 0.0001		
2400–2700 ms	*F* = 7.86, *p* < 0.0001		β = 10.73, *t* = 5.14, *p* < 0.0001		
2700–3000 ms	*F* = 12.06, *p* < 0.0001		β = 17.8, *t* = 6.79, *p* < 0.0001		

## Discussion and Conclusion

Consistent with our *a priori* hypotheses, individuals who were high vs. low in granularity showed different neural patterns during the experience of emotions at multiple time frames. This finding is made all the more interesting based on the fact that granularity was assessed as an individual difference based on daily experiences that occurred 2 days before the in-lab assessment of emotion-related ERPs and ERD/ERS. Of course, our findings are the first to examine the relationship between granularity and EEG-based measures of brain activity, so many of our hypotheses are preliminary and even exploratory in nature. Furthermore, although we situate our hypotheses in the (CAT) of emotion, the link between specific psychological mechanisms predicted by the CAT and specific ERP and ERD/ERS outcomes remain hypothetical at this point in time and certainly requires further validation. For each of the ERP and ERD/ERS findings, we discuss whether findings were consistent with our *a priori* hypotheses and offer preliminary interpretations of our findings.

### Effects of Granularity and Emotion on ERP Patterns

Our ERP analysis focused on the first 600 ms after the stimulus onset and as predicted showed impacts of granularity on the neural processing of emotion at early, middle and late stages of the stimulus presentation. These findings are in and of themselves interesting and important, insofar as granularity is an individual difference variable that was measured independently from neural responses to emotionally evocative images. This suggests that a person’s level of granularity is influencing how their brain represents emotional experiences, starting from the very initial moments of stimulus presentation.

For instance, the first significant main effect of granularity occurred between 60 and 90 ms, in which low granularity individuals showed more negative amplitudes. Little is known about this time frame, but it may reflect early attention to affectively evocative stimuli. For instance, Jiang et al. (2014) observed a greater negativity approximately 50 ms after stimulus onset, which they refer to as the N50. Of relevance to the present report, participants in Jiang et al.’s study had a greater N50 to negative vs. positive words (Jiang et al., 2014). Similarly, Hofmann et al. (2009) found greater negativity to positive and highly arousing words (as compared to neutral words) in a slightly later, yet still quite early (i.e., 80–120 ms) time frame. Both of these ERP components are earlier than the N1 and P1, which are known to respond to the affective valence and arousal of stimuli. Since little is known about this time frame, we use caution when interpreting our findings, but they could suggest that lowly granular individuals may have greater initial allocation of attention to emotionally evocative stimuli than do highly granular individuals. Although we did not predict this outcome, it is nonetheless interesting as the remainder of our ERP findings showed evidence of greater amplitudes in high granularity individuals, especially in the components related to the conceptual processing of stimuli and executive control that we predicted. Low granularity individuals may thus have early reactivity to affective stimuli, but do not then engage conceptual resources to help make meaning of their reactions to affective stimuli. This could explain why lowly granular individuals are ultimately worse at emotion regulation (Barrett et al., 2001), because they have early reactivity to evocative stimuli but then do not engage resources to help make meaning of and subsequently regulate their affective responses.

Consistent with our hypotheses, we also observed main effects of granularity during the middle stages of stimulus presentation, particularly during the negativity between 270 and 300 ms post-stimulus. This timeframe is associated with the N2 response, and individuals high in granularity had a more negative N2 amplitude than individuals low in granularity. The N2 is generally associated with “cognitive control” (Folstein and Van Petten, 2008), a concept that incorporates use of working memory, initiation of behavioral responses and inhibition of pre-potent responses. These findings suggest that highly granular individuals may be accessing working memory to a greater degree than lowly granular individuals during this early-to-middle timeframe of the emotional experience. Consistent with the CAT’s hypothesis that discrete emotions emerge in consciousness when affective reactions are made meaningful as instances of anger, disgust, fear, etc. using concept knowledge (Lindquist and Barrett, 2008a), greater N2 activity may reflect high granularity individuals’ greater use of WMC to access relevant conceptual knowledge (e.g., to access conceptual knowledge about “anger” when viewing an angry image), but also to inhibit irrelevant conceptual knowledge (e.g., to inhibit conceptual knowledge about “disgust,” “fear,” and “sadness” when viewing an angry image). Since the N2 has been associated with activity within the dACC (e.g., Luus et al., 2007), these findings also converge with Lane et al.’s (1998) findings that individuals higher in emotional awareness, another form of emotional complexity, show greater dACC activity during emotions. The fact that executive control is occurring relatively early in the stimulus presentation (around 270 ms) is consistent with the CAT’s hypothesis that granularity stems from a systematic difference in the engagement of executive control and conceptual processes during emotionally evocative events, not merely as a product of labeling after the emotion has occurred.

Finally, we observed main effects of granularity in the later (540–570 ms) range of stimulus presentation. Here, high granularity individuals again had a greater and more sustained positive amplitude than low granularity individuals, reflecting a greater LPP. The related LPP is thought to be involved in “motivated attention,” and executive control (Hajcak et al., 2009) and seems to be particularly involved when meaning is made of stimuli. For instance, the LPP is observed during emotion regulation when the emotional meaning of stimuli is reappraised as having a different meaning (Moser et al., 2006; Hajcak and Nieuwenhuis, 2006; Foti and Hajcak, 2008). Although some models conceive of a difference between emotion generation and emotion regulation (Barrett et al., 2001), the CAT does not draw firm distinctions between these processes. According to the CAT, both the initial categorization of one’s affective state, as well as later re-conceptualization during emotion regulatory strategies, involves the same basic processes: categorization. We thus predicted and found that individuals higher in granularity are recruiting attentional resources to a greater extent for the categorization of the meaning of their affective states.

By contrast to these main effects of granularity, the main effect of experiencing specific emotions influenced brain activity primarily in the later, 330–540 ms range. This range of ERPs is associated with components such as the P300, N400, and LPP, which are related to motivated attention, cognitive control, and meaning processing. These findings are consistent with the Conceptual Act Theory’s hypothesis that discrete emotions emerge in consciousness only when meaning is made of early affective (positive and negative) responses to stimuli (Lindquist and Barrett, 2008a). The fact that experiences of discrete emotions are differentiated only during this middle phase associated with access to conceptual knowledge is consistent with the constructionist hypothesis that discrete emotions (e.g., fear vs. disgust; awe vs. excitement) are constructed phenomena that are not instantly and automatically triggered by a stimulus. Rather, the Conceptual Act Theory predicts that individuals experience relatively automatic valenced affective responses early on (as indicated by the P1 and N1) that are subsequently made meaningful as fear vs. disgust or awe vs. excitement when conceptual knowledge is accessed to categorize the meaning of the affective state in late processing stages. Since we did not have *a priori* hypotheses about how emotions would differ from one another, we do not interpret the mean differences listed in **Table 2**. However, it is notable that awe and excitement were generally associated with relatively more positive amplitudes than neutral, and that fear and disgust were associated with relatively more negative amplitudes than neutral. It should be noted that although it is possible that the ERP differences are a product of the emotion category used, we cannot rule out that differences in valence and arousal are driving at least some of these ERP differences. Although we attempted to equate valence and arousal in our stimulus set (e.g., by matching awe and excitement and fear and disgust in valence), there is still a difference in positive vs. negative valence between awe/excitement and fear/disgust as well as a difference in the arousal content of our stimuli within valence. For instance, fear and excitement were on average higher in arousal than disgust and awe (see **Figure 1**). Nonetheless, this is one of relatively few studies to examine main effects of different emotion categories as induced by pictures on ERPs, and we look forward to future research replicating and extending these findings. Indeed, growing work is examining ERPs to discrete emotion categories. Other recent studies compare specific discrete emotion category words (e.g., happiness) to words referencing the broader dimensions of valence (e.g., positivity) (Briesemeister et al., 2014), compare specific discrete emotion category words (e.g., disgust) to neutral words (Ponz et al., 2013) or compare the effects of perceiving different discrete emotional facial expressions to one another (e.g., Müller, 2017; for a review of studies on the perception of discrete facial expressions for emotion, see Eimer and Holmes, 2007).

It should be noted that many of the main effects observed were qualified by interactions between granularity and emotion category. Since we did not have *a priori* predictions about these interactions, we again refrain from interpreting their direction. However, it is worth noting that these interactions occurred starting even in very early time frames and ranged until middle time frames. Generally, these interactions suggest that some emotions peaked higher or lower than other emotions depending on participants’ level of granularity and that granularity was having an early effect on how the brain processed specific emotion categories. These findings suggest that high vs. low granularity individuals may be differentially sensitive to the low-level visual cues present in the stimuli that are intended to evoke certain emotional experiences, and moreover, that this might be particularly the case for certain emotional experiences over others. One finding that may be notable is the very early (0–30 ms) negativity observed when highly granular individuals viewed disgusting images. This time frame is too early for true emotion differentiation to have occurred, but since stimuli were blocked and subsequent stimuli within a block were thus predictable to participants, these findings likely reflect highly granular individuals’ greater anticipatory responding (see Teder-Sälejärvi et al., 2002) to disgusting images. Although speculative at this point, these findings imply that highly granular individuals could have greater attention to emotionally evocative stimuli than individuals lower in granularity, especially when they know to expect such images. Such a finding could again be consistent with the fact that individuals high in granularity are better at emotion regulation than individuals low in granularity (Barrett et al., 2001). When they know to expect emotionally evocative stimuli, high granularity individuals may be more likely to allocate attentional processes in order to anticipate their emotion regulation needs. Highly granular individuals also responded differently than lowly granular individuals to exciting and fearful images at other early- to middle-range timeframes. We look forward to future research that replicates and extends these findings.

### Effects of Granularity and Emotion on ERD/ERS

We used ERD/ERS to describe the oscillations in populations of neurons for a longer time frame (∼3 s) compared to the ERP analysis (∼600 ms). Alpha oscillation generally decreased over participants (alpha ERD), and lowly granular participants experienced greater alpha ERD across all emotions as compared to highly granular individuals. We predicted that individuals high in granularity would show less alpha ERD and participants low in granularity would show more alpha ERD during emotional experiences. This finding is consistent with previous studies that individuals high in emotional intelligence showed less alpha ERD than individuals who are average in emotional intelligence, when naming the meaning of facial expressions (Jaušovec et al., 2001; Jaušovec and Jaušovec, 2005). As greater ERD has been associated with attention that enables “controlled knowledge access and semantic orientation,” one interpretation of this result is that lowly granular people may have had to work harder to access conceptual knowledge to make meaning of affective stimuli. By contrast, the process of accessing concept knowledge to make meaning of an affective state may be relatively automatic for highly granular individuals. More broadly, this interpretation is consistent with a neural efficiency account (Grabner et al., 2006), which argues that more intelligent people require less cortical activation to perform well on a psychological task than do less intelligent people. For instance, highly intelligent people had less upper alpha ERD on trials from the RAVEN intelligence task that were relatively easy; less intelligent individuals had higher ERD on the same easy trials (Doppelmayr et al., 2005). By contrast, the difference in ERD between highly intelligent people and lowly intelligent people was less pronounced on difficult RAVEN trials. The neural efficiency account may thus describe why high granularity individuals had less alpha ERD during experiences of emotion. Highly granular individuals, who may be more emotionally complex, may routinely construct experiences of discrete emotion out of affective experiences, whereas lowly granular individuals do not. In other words, experiencing emotions in a discrete manner could be habitual and “easy” for high granularity people, whereas lowly granular individuals find the task more difficult. It is often thought that the so-called “cognitive” processes involved in intelligence are distinct from the so-called “emotional” processes involved in the construction of emotional experiences, but there is reason to believe that processes such as semantic retrieval and WMC more generally are implicated in the construction of emotion experiences (Barrett et al., 2004; Lindquist and Barrett, 2012). We furthermore argue that WMC ultimately limits granularity, insofar as individuals who are high in granularity must be able to access and use conceptual knowledge to make meaning of their affective states as discrete emotions in the moment. Individuals who are low in WMC, regardless of the complexity of their conceptual knowledge, should not be able to flexibly use said knowledge (see Lindquist and Barrett, 2008b). The neural efficiency hypothesis would thus suggest that individuals high in granularity may be relatively more efficient at conceptualizing the meaning of their affective states than individuals low in granularity, meaning that their brain has to work less hard to construct emotional experiences. Our findings are consistent with this account.

Our gamma ERS result conformed to the alpha ERD results. Increase in gamma ERS power can denote more effort in feature integration, attention, stimulus selection, integration of sensory inputs and sensorimotor activities, movement preparation, and memory formation (Knyazev, 2007); lowly granular individuals showed greater gamma ERS as compared to highly granular individuals between 900 and 1200 ms during the emotional experience. Interaction with discrete emotions signified such effects. These findings may again imply that lowly granular individuals had to allocate relatively more brain resources to make semantic meaning of their affective responses. Relatively stable gamma ERS of high granularity people may also fit the interpretation that experiencing emotions in a discrete manner is habitual for high granularity people. Of note, our gamma band findings are opposite to Jaušovec and Jaušovec (2005), who found that individuals high in emotional intellience had greater gamma band ERS than individuals who were average in emotional intelligence. However, Jaušovec and Jaušovec (2005) themselves note that their findings were inconsistent with their own alpha frequency findings as well as other evidence supporting the neural efficiency account, so their gamma findings may be anomoulous. Our gamma ERS findings are consistent with our alpha ERD findings, Jaušovec and Jaušovec (2005)’s alpha ERD findings, as well as our predictions about neural efficiency for emotional processing in high vs. low granularity individuals.

Emotion category and the interaction of granularity and emotion did not predict differences in alpha ERD, but we did observe main effects of emotion and interactions between granularity and emotion for the gamma ERS findings. We did not have *a priori* hypotheses about any of these findings, so we refrain from drawing strong interpretations. Nonetheless, our ERS findings, like our ERP findings, preliminarily suggest that the degree to which a person characteristically draws on conceptual knowledge of emotion interacts differently with the specific emotion content being experienced.

In sum, our findings are the first to use EEG to test predictions of the Conceptual Act Theory, and the first to examine differences in neural processes of individuals high and low in granularity during emotions. Of course, there are important limitations inherent in our study that should be noted. For one, we are taking for granted that affective stimuli induce emotional experiences and that we are measuring neural reactions to emotional experiences as opposed to the mere perception of stimuli. This is an assumption not unique to our study; it is implicit in many studies that attempt to evoke emotion in the lab using visual, auditory, or memory-based inductions. However, our assumption is bolstered by meta-analyses linking image-viewing to self-report and physiological changes indicative of emotional experiences (e.g., Lench et al., 2011) and norming studies linking specific types of images to the experience of specific emotions (e.g., Mikels et al., 2008). We are also drawing inferences that certain EEG outcomes are indicative of the presence of certain psychological processes (i.e., executive control) during emotions. We recognize that neuroimaging-based methods such as EEG are ultimately correlational and cannot provide inferences about causation. Neuroimaging-based methods are also subject to the reverse inference problem (Poldrack, 2006), in which the engagement of a particular mental function is inferred from the presence of certain neural activity. However, the fact that we predicted the involvement of certain processes (e.g., executive control) in emotions *a priori* begins to mitigate this concern. Ultimately, converging evidence from other methods such as lesion-based approaches can help confirm whether hypothesized processes are involved in emotions and whether they are sufficient or necessary.

Although preliminary, we believe our findings offer important initial evidence that can spur future research. For instance, findings from this research have important theoretical consequence, as they can begin to weigh in on the temporal dynamics of the neural processes involved during emotion, as well as the temporal dynamics that are influenced by an emotion-relevant individual difference (i.e., emotional granularity). Our findings are of also of practical use in more applied domains, such as in brain-computer interfaces in which neuroadaptive systems attempt to use neurophysiological signals indexing the user’s emotions to cause changes in functional characteristics of the system. A neuroadaptive system can be used to provide feedback to users based their emotional status while using the system (Hettinger et al., 2003), but must ultimately be sensitive to the individual differences that characterize emotion. As we’ve shown here, granularity might be one such important individual difference to consider in future applied research.

## Ethics Statement

North Carolina State University Human participants were given a detailed explanation of the experiment and signed on the consent form when they agreed to participate. General population without any disabilities participated in the study.

## Author Contributions

All authors listed, have made substantial, direct and intellectual contribution to the work, and approved it for publication.

## Conflict of Interest Statement

The authors declare that the research was conducted in the absence of any commercial or financial relationships that could be construed as a potential conflict of interest.
